# Account of Diseases in an Eastern District of London, from the 20th of June to the 20th of July, 1800

**Published:** 1800-08

**Authors:** 


### STATE OF DISEASES IN LONDON.

*Account of Diſeaſes in an Eaſtern Diſtrict of London, from the* 20*th of June to the* 20*th of July,* 1800.

| | No. of Caſes. | | No. of Caſes. |
|---|---|---|---|
| ACUTE DISEASES. | | Dyſentery - - - - - - | 3 |
| Typhus mitior - - - - - | 7 | CHRONIC DISEASES. | |
| Typhus gravior - - - - | 3 | Phthiſis Pulmonalis - - - - | 3 |
| Small-Pox - - - - - - | 7 | Cough with Dyſpnœa - - | 6 |
| | | Hæmoptoe | |

| | |
|---|---|
| Hæmoptoe | 3 |
| Hydrothorax | 4 |
| Gaſtrodynia | 3 |
| Dyſpepſia | 10 |
| Diarrhœa | 16 |
| Colica Pictonum | 1 |
| Aſcites | 6 |
| Anaſarca | 4 |
| Prolapſus Ani | 1 |
| Inteſtinal Hæmorrhagy | 2 |
| Cephalalgia | 8 |
| Vertigo | 5 |
| Hemiplegia | 3 |
| Epilepſy | 4 |
| Pſora | 2 |
| Tinea | 2 |
| Herpes Puſtuloſus | 1 |
| Chronic Rheumatiſm | 10 |
| PUERPERAL DISEASES. | |
| Low Puerperal Fever | 2 |
| Menorrhagia lochialis | 3 |
| Iſchuria | 1 |
| INFANTILE DISEASES. | |
| Diarrhœa | 6 |
| Watery Gripes | 3 |
| Aphthæ | 4 |
| Scrophula | 2 |

One of the caſes of ſmall-pox referred to in the liſt, was that of a pregnant woman, upon whom the puſtulary eruption appeared a few days previouſly to her labour. The diſeaſe was of the confluent kind, and, as might be expected, in a few days after her delivery, it terminated fatally. The child was born without any marks of infection; but on the fourth day after its birth, an eruption appeared on the face and neck, and on different parts of the body, which proceeded in the uſual way to form puſtules, of which there was a conſiderable number, and in a few days it fell a victim to the diſeaſe.

A variety of opinions has been formed reſpecting the effect of the variolous infection on the fœtus in utero; a ſufficient number of inſtances, however, has been recorded, to aſcertain that the diſeaſe may be communicated from the mother to the child. In ſome caſes, the body of the child, at its birth, has been covered with puſtules, and the nature of the diſeaſe has been moſt ſatisfactorily aſcertained by inoculating with matter taken from the puſtules.

In other caſes, as in the preſent inſtance, there has been no appearance of the diſeaſe at the time of the birth; but an eruption and other ſymptoms of the diſeaſe have appeared ſo early as to aſcertain that the infection muſt have been received previouſly to the removal of the child from the uterus. Thus, as Dr. Meade obſerves, the ſmall-pox which the infant has contracted in the womb, breaks forth on the ſecond, third, or any other day before the 8th from the delivery.

MONTHLY

